# Complications of Cardiac Resynchronization Therapy: Comparison of Safety Outcomes from Real-world Studies and Clinical Trials

**DOI:** 10.19102/icrm.2022.130805

**Published:** 2022-08-15

**Authors:** Naga Venkata K. Pothineni, Suhas Gondi, Tharian Cherian, Swathi Kovelamudi, Robert D. Schaller, Dhanunjaya Lakkireddy, Rakesh Gopinathannair, Abhishek Deshmukh

**Affiliations:** ^1^Division of Cardiology, University of Pennsylvania, Philadelphia, PA, USA; ^2^Harvard Medical School, Boston, MA, USA; ^3^Divison of Cardiology, University of Arkansas, Little Rock, AR, USA; ^4^Kasnas City Heart Rhythm Institute, Overland Park, KS, USA; ^5^Division of Cardiology, Mayo Clinic, Rochester, MN, USA

**Keywords:** Cardiac resynchronization therapy, complications, registries

## Abstract

Cardiac resynchronization therapy (CRT) is an important intervention in heart failure. Whether real-world complication rates mirror those reported in randomized clinical trials (RCTs) is unknown. We sought to compare rates of procedural complications between major RCTs of CRT with “real-world” complication rates reported in registries and administrative claims database studies. We conducted a PubMed search to identify all relevant publications on CRT and classified them into RCTs and registry studies. Pooled procedural complication rates were analyzed. Differences between groups were compared using the chi-squared test. We identified a total of 6 RCTs, 2 administrative claims database studies, and 4 CRT registry studies. RCTs included a total of 4,442 patients and “real-world” studies included a total of 72,554 patients. The overall rates of procedural complications with CRT were significantly higher in RCTs compared to the real world (8.1% vs. 6.9%, *P* = .002). Lead-related complications were higher in the real-world studies compared to RCTs (11.3% vs. 6.5%, *P* = .0001). This could represent a follow-up bias with patients in registries being followed up for longer durations that would compound lead complication rates. Interestingly, RCTs had a higher incidence of pocket hematomas (2.1% vs. 0.4%, *P* = .001). In conclusion, real-world procedural complication rates of CRT appear to be significantly lower than those reported in RCTs.

## Introduction

Cardiac resynchronization therapy (CRT) has revolutionized the treatment of heart failure with reduced ejection fraction and ventricular dyssynchrony.^1^ Over the last decade, the utilization of CRT has dramatically increased, with a major rise in implant rates all over the world.^2^ However, CRT implantation is not without risks and requires an individual risk–benefit assessment, based primarily on published outcome and complications data from randomized controlled trials (RCTs). In addition to forming the basis for a risk–benefit analysis, these risk estimates and reported complication rates are often used as a surrogate marker for operator and institutional performance and may soon be linked to reimbursement. However, previous studies have shown discrepancies between the complication rates of various cardiovascular procedures in real-world settings and those in RCTs. For instance, the rate of implantable cardioverter-defibrillator (ICD) complications reported in the National Cardiovascular Data Registry–ICD registry (NCDR-ICD) was almost 3-fold lower than that found in RCTs.^3^ Similar differences have been reported in complications of percutaneous catheter ablation as well.^4^ We sought to estimate and compare the pooled rates of procedural complications between major RCTs of CRT and “real-world” complication rates reported in registries and administrative claims database studies.

## Methods

### Literature review

A PubMed search was conducted from inception until December 2017 using the keywords “cardiac resynchronization therapy” and “complications.” All abstracts that reported in the English language were reviewed. Both prospective and retrospective studies were included, while meta-analyses and review articles were excluded. The resulting articles were manually reviewed, and full-length articles that reported complication rates of CRT were included in the study. Those studies on congenital heart disease, CRT upgrades, generator changes, and ICD implantation were excluded.

### Data extraction

The selected articles were reviewed by 3 investigators (N. V. K. P., S. K., and S. G.) and were classified into RCTs, registries, and administrative database studies. Relevant patient characteristics, sample sizes, and individual procedural complications were extracted and recorded. Reported complications were classified into the following categories: lead-related (lead failure/dislodgement, need for lead revision/replacement, lead fracture, lead undersensing, T-wave oversensing, insulation failure), access-related (hematoma, pneumothorax), infection, mortality, and overall complications.

### Statistical analysis

We summarized categorical variables as counts and percentages and continuous variables as mean and range values. We used Microsoft Excel for Mac 2017, version 15.30 (Microsoft Corporation, Redmond, WA, USA) to create clustered column charts and tables. We used the chi-squared test, Wilcoxon rank-sum test, and Fisher’s exact test as appropriate to compare between-group differences. *P* < .05 was considered to indicate statistical significance.

## Results

Our search strategy yielded a total of 1,126 full-length articles. After individual review, 15 full-length articles that reported CRT complication rates were identified, of which 6 were RCTs and 5 were device registry studies. Four articles used data from administrative claims databases of the Nationwide Inpatient Sample (NIS) and NCDR-ICD. The characteristics of these studies are described in **Table 1**. A total of 4,465 CRT implants were included in the RCTs, whereas 175,045 implants were included in the registries and administrative claims databases. The follow-up duration among RCTs ranged from 6–40 months.

### Randomized clinical trials

Major randomized trials of CRT implantation that were included in our analysis were Multicenter InSync Randomized Clinical Evaluation (MIRACLE); Comparison of Medical Therapy, Pacing, and Defibrillation in Heart Failure (COMPANION); Cardiac Resynchronization—Heart Failure (CARE-HF); Resynchronization Reverses Remodeling in Systolic Left Ventricular Dysfunction (REVERSE); Multicenter Automatic Defibrillator Implantation Trial with Cardiac Resynchronization Therapy (MADIT-CRT); and Resynchronization–Defibrillation for Ambulatory Heart Failure Trial (RAFT) and included a total of 4,465 CRT implants **(Table 1)**. The follow-up durations ranged from 6–40 months with a mean follow-up duration of 24 months, and all RCTs were conducted prior to 2010. There were no data for complications available in the COMPANION trial. MIRACLE reported 2 cases of death and 7 lead-related complications. Among all the RCTs, only MIRACLE and CARE-HF reported any mortality data related to CRT implantation. The most common procedural complications reported among RCTs were lead-related (6.5%), followed by hematoma (2.0%), infection (1.5%), pneumothorax (1.3%), and death (0.5%). There were a total of 361 overall complications amounting to a complication rate of 8.1% **(Table 1)**.

### Registries

Five major CRT registries that included 12,266 CRT implants from 2003–2013 were analyzed. The most common complication reported was lead-related (3.52%), followed by hematoma (2.54%), infection (1.03%), pneumothorax (0.59%), and death (0.22%), with an overall complication rate of 6.26% **(Table 1)**. The Management of Atrial fibrillation Suppression in AF–Heart Failure Comorbidity Therapy (MASCOT) registry only reported 5 cases of infection, and data on other complications were not described. The Danish registry reported a high incidence of lead-related complications (15.1%) in a relatively small patient population of 654. Data on other types of complications were not available in the Danish registry. Only the French and the German registries reported any incidence of death related to CRT implantation (10 and 5 deaths, respectively).

### Administrative databases

Four studies were identified that reported complication rates of CRT within a study period of 2007–2014. Two studies used the NIS, while the other 2 included data from the NCDR-ICD with a total number of 162,779 CRT implants. The incidence of lead-related complications was 6.75%, followed by pneumothorax at 1.44%. Other complications included pocket hematoma (1.31%), infection (1.28%), and death (0.78%). The overall complication rate among implants reported in these administrative databases was 5.57% **(Table 1)**. The NCDR database only yielded data on infection and lead-related complications, whereas NIS did not capture any lead-related complications.

### Randomized controlled trials versus the real world

**Figure 1:**
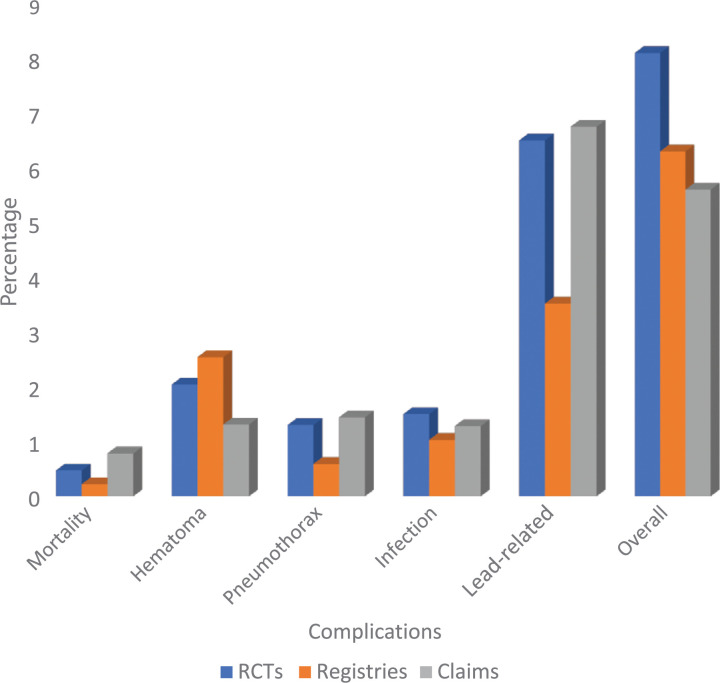
Rates of reported complications of cardiac resynchronization therapy in randomized controlled trials, registries, and administrative databases. *Abbreviation:* RCTs, randomized controlled trials.

**Figure 2:**
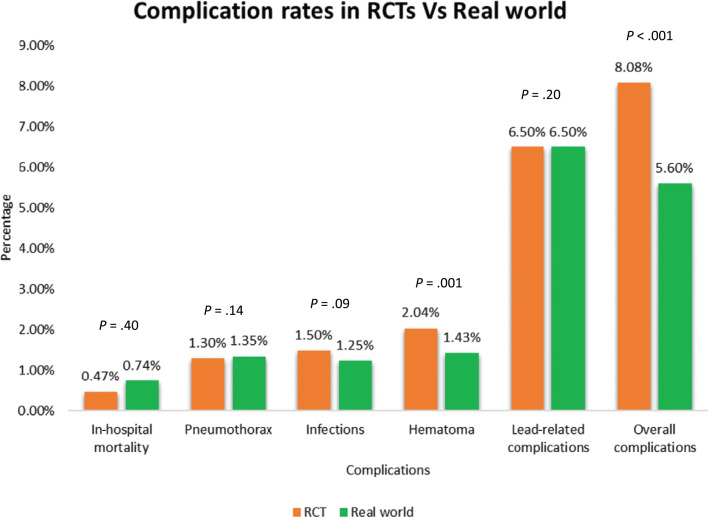
Bar chart depicting complication rates of cardiac resynchronization therapy implants in randomized controlled trials versus real-world studies along with *P* values for between-group differences. *Abbreviation:* RCT, randomized controlled trial.

A comparison of individual complication rates between RCTs, registries, and administrative studies is presented in **Figure 1**. Data from registries and administrative studies were pooled to reflect a “real-world” sample. This comparison of complication rates between RCTs and real-world studies demonstrated significantly lower overall complication rates in real-world studies than those reported in RCTs (5.6% vs. 8.1%, *P* < .001) **(Figure 2)**. Hematomas were reported less often in the real-world databases than RCTs (1.4% vs. 2.0%, *P* = .001). There was also a trend toward a lower rate of infection in the real-world setting (1.5% vs. 1.25%, *P* = .09) than RCTs.

## Discussion

In this systematic review of CRT complications, we report a significant discrepancy in complication rates of CRT between randomized trials and “real-world” outcomes. The overall complication rate of 8.1% in RCTs was significantly greater than the 5.6% complication rate in the real world, as estimated by aggregation of data from registries and administrative claims databases. Rates of all individual procedure-related complications were similar between RCTs and real-world studies, except for the incidence of pocket hematomas, which was higher in RCTs (2.04% vs. 1.43%, *P* = .001).

There are several potential explanations for these findings. Given that the uptake of procedures like CRT in the real world often follows major RCTs that validate their safety and efficacy, it is possible that the decreased complication rate in the real world simply reflects the increasing operator experience and technical improvements over time. Better operator training and improved procedural techniques are expected with the evolution of CRT as a mainstream treatment for heart failure. This explanation presumes that the publication of the RCT data predates the real-world data, which is generally the case in this study: the RCTs span 2002–2010, while the registry studies span 2012–2017 and the administrative claims database studies span 2007–2017. As such, the decreased complication rate may be the result of time and experience with the procedure. However, there was some overlap in time when considering the study periods during which data were collected. One of the registry studies (MASCOT) had a study period of 2003–2006 and another (French) included 2002–2010 in its study period, while 2 others (Kaiser Permanente and German) included 2007–2010. As for the administrative databases, the study periods all overlap with the RCTs: 1997–2004, 2006–2009, 2006–2010, and 2003–2014. Given these overlaps, it is unlikely that increased experience with the procedure can fully explain the disparity in aggregate CRT complication rates.

Another potential explanation for the difference may be a lack of standardized definitions for reporting complications. Each study, whether an RCT, a claims-based study, or a registry-based study, has its own definition and reporting system for various complications. In the context of CRT, this could significantly impact the reported rates of lead complications and infections due to variability in how these events may be adjudicated. This inconsistency may explain, in part, the disparity we observed and the calls for greater standardization of these definitions across the field. Differences in complication rates observed could also be related to follow-up time periods between RCTs and the real world. Some complications, such as pneumothorax, present immediately, either during the CRT implant or shortly thereafter. Others, such as pocket infection or a lead integrity issue, may only present days to months after discharge. As claims and registries provide point source data, they are less likely to accurately capture the frequency of late-onset complications. This may explain in part why the incidence of pocket hematomas was greater in the RCTs than in the real world. A final explanation for our results may be that under-reporting and/or under-recognition of complications is occurring in the real world. Prior studies have raised concern about possible under-reporting of complications for other electrophysiology procedures, such as ICD implants.^5–7^ This possibility ought to be considered given the ongoing shift from a fee-for-service system toward a more value-based system.^8^

The disparity in the reported complication rates of CRT between RCTs and real-world data is worrisome and could influence patient care by quoting inaccurate complication rates depending on the source of their data. It is critical for the cardiology community to study the disparity in complication rates of all invasive procedures between RCTs and the real world to enable translation of evidence-based care. In addition to the importance of informed patient consent, the need for more research on this topic is intensified by the sector-wide movement to measure procedural outcomes and base physician and hospital reimbursement on performance.^9^ Standardization of definitions and protocols around reporting is a prerequisite to understanding this problem further. Professional societies, payers, and policymakers should collaborate in determining and promulgating such standards. The National Quality Forum has already begun this important work for the field of electrophysiology.^10^

There are a number of limitations to this study. First, variations in reporting complication rates could have influenced the results of the study. Second, several confounding factors—such as procedural techniques (vascular access), patient comorbidities, antibiotic regimens, and patient selection for the procedure—cannot be accounted for in this analysis. Third, the real-world data are predominantly taken from the NIS database, which captures only a portion of all CRTs and only include in-hospital complications. Fourth, the lengths of follow-up varied across the different studies, meaning some complications that arise late in the post-procedural period may have been more likely to be captured in certain studies than others. Lastly, the retrospective nature of this study is inherently limiting.

## Conclusion

CRT continues to evolve as an essential treatment modality for patients with heart failure and ventricular dyssynchrony and to prevent sudden cardiac death. Nevertheless, the variations of outcomes in RCTs and registries warrant reforms to standardize definitions and reporting of complications to ensure accurate and reliable public reporting of complications and outcomes. This calls for increased advocacy to implement better data collection methods to accurately capture overall complication rates.

## Figures and Tables

**Table 1:** Complications of CRT Implants in Various Studies

Studies	Follow-up (Months)	Number of Implants	Mortality	Pneumothorax	Hematoma	Infection	Lead-related
RCTs							
MIRACLE	6	228	2	NA	NA	NA	7
COMPANION	16	1,080	NA	NA	NA	NA	NA
CARE-HF	29	390	1	6	NA	3	24
REVERSE	12	621	NA	4	5	NA	66
MADIT-CRT	29	1,007	NA	19	36	12	NA
RAFT	40	841	NA	11	14	21	61
Total		4,465	0.47%	1.3%	2.04%	1.5%	6.5%
Registries	Study period (years)						
MASCOT	2003–2006	402	NA	NA	NA	5	NA
Danish	2010–2011	654	NA	NA	NA	NA	99
French	2002–2012	5,539	10	44	253	54	195
KP	2007–2013	4,472	NA	6	17	49	NA
German	2007–2011	1,199	5	16	15	NA	NA
Total		12,266	0.22%	0.59%	2.54%	1.03%	3.52%
Claims database							
NIS-2007	1997–2004	8,261	77	78	23	22	NA
NCDR-ICD1	2006–2009	3,545	NA	NA	NA	224	404
NCDR-ICD2	2006–2010	58,493	NA	NA	NA	NA	3,789
NIS-2017	2003–2014	92,480	703	1,369	1,304	1,082	NA
Total		162,779	0.78%	1.44%	1.31%	1.28%	6.75%

*Abbreviations:* CARE-HF, Cardiac Resynchronization—Heart Failure; COMPANION, Comparison of Medical Therapy, Pacing, and Defibrillation in Heart Failure; CRT, cardiac resynchronization therapy; KP, Kaiser Permanante; MADIT-CRT, Multicenter Automatic Defibrillator Implantation Trial with Cardiac Resynchronization Therapy; MASCOT, Management of Atrial fibrillation Suppression in AF-HF Comorbidity Therapy; MIRACLE, Multicenter InSync Randomized Clinical Evaluation; NA, not available; NCDR-ICD, National Cardiovascular Data Registry–ICD registry; NIS, Nationwide Inpatient Sample; RAFT, Resynchronization–Defibrillation for Ambulatory Heart Failure Trial; RCT, randomized controlled trial; REVERSE, Resynchronization Reverses Remodeling in Systolic Left Ventricular Dysfunction.
